# Consequences of tumor planning target volume reduction in treatment of T2-T4 laryngeal cancer

**DOI:** 10.1186/1748-717X-9-195

**Published:** 2014-09-04

**Authors:** Cornelia AJM Vugts, Chris HJ Terhaard, Marielle EP Philippens, Frank A Pameijer, Nicolien Kasperts, Cornelis PJ Raaijmakers

**Affiliations:** Department of Radiotherapy, Academic Medical Center, Meibergdreef 9, 1105 AZ Amsterdam, The Netherlands; Department of Radiotherapy, University Medical Center Utrecht, Utrecht, The Netherlands

**Keywords:** Laryngeal carcinoma, Margin reduction, NTCP

## Abstract

**Background and purpose:**

Since lymph nodes volumes are generally four times the volume of the primary PTV, the advantage of using tight margins around the primary PTV is not clear. Therefore treatment margins of T2-T4 laryngeal carcinoma for IMRT are generally chosen in such a way that the PTV is comparable to that in conventional radiotherapy. The aim of this study is to quantify the effect of volume reduction of the primary PTV of T2-T4 laryngeal carcinoma with regard to late toxicity despite elective irradiation of lymph node levels II to IV.

**Methods:**

Two treatment plans based on conservative (GTV-PTV = 15 mm and 20 mm cranial), and on evidence-based tight margins (GTV-PTV = 8 mm) were calculated for 16 patients. Toxicity effects were estimated based on the dose distributions.

**Results:**

Compared to conservative margins, using tight margins resulted in: 1) significant reduction of the normal tissue complication probability (NTCP) for swallowing muscles and submandibular glands, 2) significant reduction of the mean dose in all organs at risk (OAR), 3) a mean dose smaller than 60 Gy for all OARs except for the laryngeal cartilages. When the lymph node levels II to IV were prescribed with an elective dose, an NTCP reduction of 53% for the swallowing muscles and of 23% for the submandibular glands was found by using tight instead of conservative margins. When positive nodes were present, NTCP reduction amounted to 29% and 15%, respectively.

**Conclusions:**

There is a potential benefit in realizing evidence-based tight margins for laryngeal cancer patients despite elective irradiation of lymph node levels II to IV.

## Background

The tumor control rate for advanced laryngeal carcinomas is significantly improved using accelerated radiotherapy. However, treatment intensification can be accompanied by severe late toxicity [1–3]. Generally, less toxicity is expected using reduced treated volumes [4–6].

Planning target volumes (PTV) can be reduced using intensity modulated techniques. These techniques require delineation of the gross target volume (GTV) and an expansion of this volume according to ICRU 62 [7]. Today, treatment margins of laryngeal carcinomas when applying intensity modulated techniques are generally chosen in such a way that the PTV is comparable to that in conventional radiotherapy. This means that the complete cartilage skeleton of the larynx is defined as target volume [8–10]. Clear guidelines for margins to define the clinical target volume (CTV) and the PTV are not available yet [11]. Perez stated: ‘delineating the CTV is more an art than a science because current imaging techniques are not capable of detecting subclinical tumor involvement directly’ [12].

Although tumor PTV reduction will apparently result in decreased toxicity, this is not obvious in case of laryngeal cancer including elective irradiation of lymph node levels II to IV. The volume of the lymph nodes is generally four times the volume of the adjacent PTV. The aim of this study was to quantify the effect of volume reduction of the primary PTV of T2-T4 laryngeal carcinoma with regard to late toxicity despite elective irradiation of lymph node levels II to IV. The effect on late toxicity is analysed using the mean dose and the normal tissue complication probability (NTCP) of organs at risk (OARs) from treatment plans based on tight and conservative margins.

## Methods

### Patients

The research was carried out in compliance with the Helsinki declarations. Sixteen patients with T2–T4 supraglottic laryngeal carcinomas were included in this planning study (Table 1). They were divided in two subgroups based on TNM-stage. Group 1 included patients prescribed with an elective dose to the volume containing lymph node levels II to IV next to a high dose to the primairy tumor (n = 10). Group 2 included patients prescribed with a high dose to the primairy tumor and to the positive lymph nodes and with an elective dose to the whole volume of lymph node levels II to IV (n = 6).

**Table 1:** **Patient characteristics**

***Patient***	***TNM-stage***	***GTV [cm*** ^***3***^ ***]***	***PTV***_***clinical***_ ***[cm*** ^***3***^ ***]***	***PTV***_***tight***_ ***[cm*** ^***3***^ ***]***
**Group 1**	**Patients without positive nodes**			
	T3N0M0	6.6	92.6	49.3
	T3N0M0	10.2	126.5	44.7
	T2N0	8.1	105.1	51.9
	cT2N0	3.1	76.5	34.4
	cT2N0M0	7.1	78	39.8
	cT3N0M0	2.3	47.9	21.4
	T2N0	4.4	38.2	26.7
	T4aN0M0	26.2	145.9	92.8
	T3N0	6.9	82.5	44.4
	T3N0M0	14.8	122.9	66.5
**Group 2**	**Patients with positive nodes**			
	T4N2c	20	96.7	83.6
	T2N2cM1	9.3	30.8	20.7
	T3n2cM0	20.6	166.4	98.7
	T2N2xM1	9.1	100.8	52.9
	T2N1	8.7	98.1	44.1
	cT2N2bM0	9	110.7	57.6
**Average**		10.4	95.0	51.8

### Delineations

**Figure 1:**
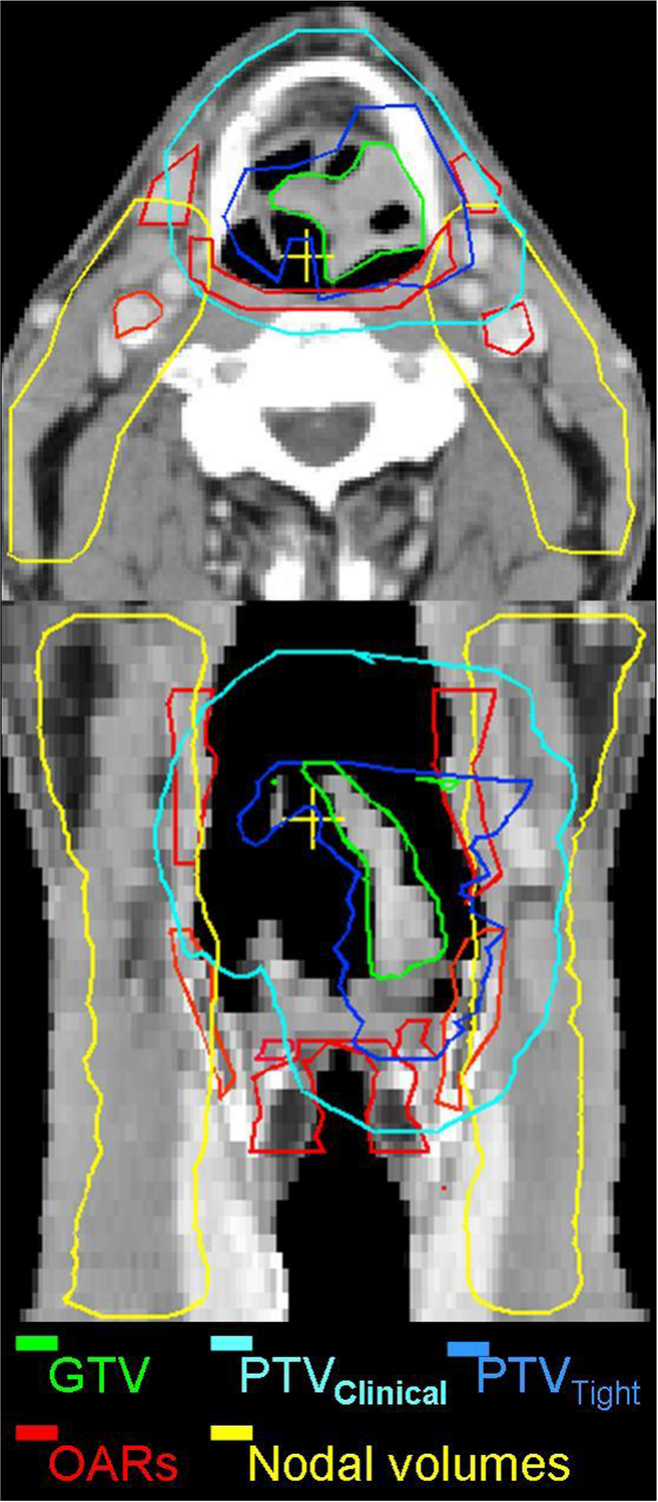
**Target volumes and OARs on a transverse (upper) and a coronal (lower) slice of a laryngeal cancer patient.**

Target structures and OARs were delineated on the planning CT scan with a slice thickness of 2 mm. Delineations were performed manually using in-house developed software. In order to account for interobserver variation, the primary tumor was delineated independently by two experienced head-neck radiation oncologists and one radiologist. The GTV was defined by union of these delineations. Lymph node levels II to IV were delineated according to the guidelines of Levendag et al. [13]. Delineated OARs included swallowing muscles, base of tongue, mucosa, submandibular glands, arytenoids, cricoid - and thyroid cartilage and carotid arteries. For delineation of the OARs, a registered MRI scan in mask (gadolinium contrast enhanced T1 weighted, fat-suppressed, MRI) was used. The SM were delineated as one structure extending from the oropharynx to the inlet of the esophagus. All tissue extending less than 2 mm from the trachea, situated between the uvula and the C6 vertebra, was defined as being mucosa (auto threshold grey value of -300 HU). The carotid arteries were contoured from the uvula to the lung apex. Care was taken to ensure that the different target volumes did not extend within 4 mm of the skin, to avoid PTV extension within the build-up region, unless the tumor extended less than 5 mm underneath the skin (Figure 1). CTV, internal target volume (ITV) and PTV were obtained by the expansion of the GTV using: 1) clinically applied, conservative margins and 2) evidence based tight margins [1, 7]. Anatomical margins were corrected for anatomical boundaries and air.

### Margins and dose prescription

The PTV of the nodal volumes resulted from a 5 mm margin around the delineated volume and were prescribed with a dose of 47 Gy (EQD_2Gy_ = 45.9 Gy, α/β = 3 Gy). The primary target volumes were prescribed with a dose of 69.5 Gy (EQD_2Gy_ = 66.2 Gy, α/β = 3 Gy).

Clinically, the GTV was extended with a CTV-margin of 15 mm and another 5 mm in cranial direction to correct for swallowing induced tumor motion [14, 15]. The CTV was extended with a uniform PTV-margin of 5 mm to result in the PTV (PTV_clinical_).

**Figure 2:**
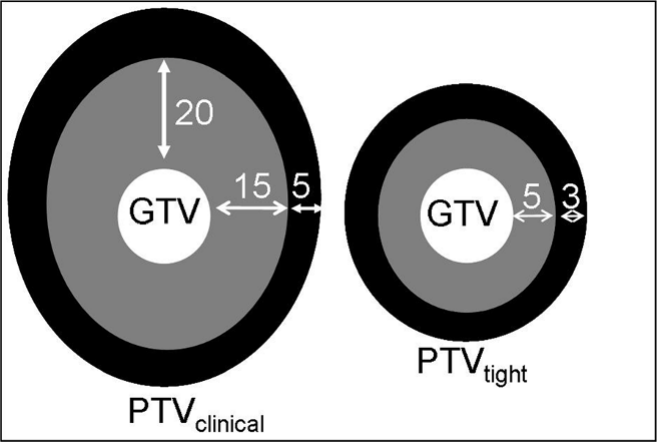
**Margin definition.**

The PTV, resulting from using tight margins (PTV_tight_) is defined by a uniform CTV-margin of 5 mm around the GTV and an uniform PTV-margin of 3 mm around the CTV (Figure 2). These tight margins can be used without comprising the treatment of the GTV since:

- Recent studies indicate an over estimation of the GTV delineated on the planning CT scan of more than 3.5 mm [16, 17].
- Preliminary results of a study in which GTV delineations are validated with pathology showed that there is in 10 out of 15 patients no microscopical spread beyond 5 mm of the GTV delineated on a CT scan [18].
- The incidence and total duration of swallowing is low. Using adaptive radiotherapy with respect to intrafraction tumor motion, internal margins can even be omitted [14].
- Mean maximal GTV displacements (not swallowing induced) are smaller than 3 mm [15].
- Improved stabilization materials in combination with CBCT for daily image guidance can result in smaller PTV margins. Systematic and random set-up errors smaller than 1 mm have been reported for vocal cord irradiation [19, 20].

### Fractionation

The dose fractionation was planned according to our clinically used accelerated concomitant boost protocol [1]. The first two weeks, five fractions of 2 Gy and in the last three weeks, ten fractions of 1.5 Gy in the morning and 1.8 Gy in the afternoon were prescribed. The time between two fractions is at least 6 hours.

### Treatment planning

For each patient, one intensity modulated radiotherapy (IMRT) plan based on conservative margins and one based on tight margins was generated. Treatment plans were created with Monaco 3.0 (Elekta/CMS, Sweden, Stockholm). The total dose distribution resulted from the addition of two plans: a plan for the volumes with a prescribed dose of 47 Gy and a supplementary plan of 22.5 Gy for the volumes with a prescribed dose of 69.5 Gy. The same template with seven beam angles was applied in all plans.

### NTCP calculation

The effect of the margin reduction on the dose to the swallowing muscles and the submandibular glands was qualified by calculating the NTCP. A dose response model of Jackson et al. based on 428 patients was used to determine the NTCP for grade 2 swallowing dysfunction. This model is based on the total volume of the swallowing muscles (α = 1.6 Gy^-1^, γ_50_ = 2.6, EUD_50_ = 80.2 Gy) [21].

For the submandibular glands, a NTCP curve of Dijkema et al. was used (TD_50_ = 35.0 Gy, m = 0.44). A complication was defined as a flow of less than 25% of the pre-treatment flow [22].

### Statistics

Wilcoxon rank test for related samples was applied for comparison of median values using an alpha level of 0.05 (SPSS inc., Chicago, USA).

## Results

### Target volume

Volume analysis yielded a mean GTV of 10.4 cm^3^. PTV_clinical_ had a mean volume of 95.0 cm^3^ and PTV_tight_ of 51.8 cm^3^ (Table 1).

### Dose distribution

**Figure 3:**
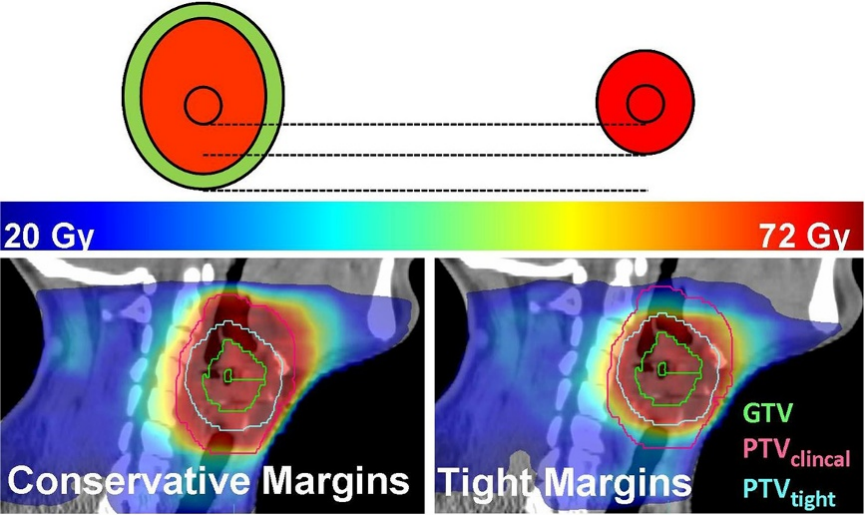
**Sagittal view of a typical dose distribution, schematic (upper) and calculated (lower), resulting of using clinical (left) and tight margins (right).**

In general, dose distributions were obtained that met our criteria: 95% of the PTV was at least covered with 95% of the prescribed dose. The spinal cord was spared in all plans with a maximum dose of 40 Gy. The volume that received over 105% of the prescribed dose was smaller than 3 cm^3^ (Figure 3).

**Figure 4:**
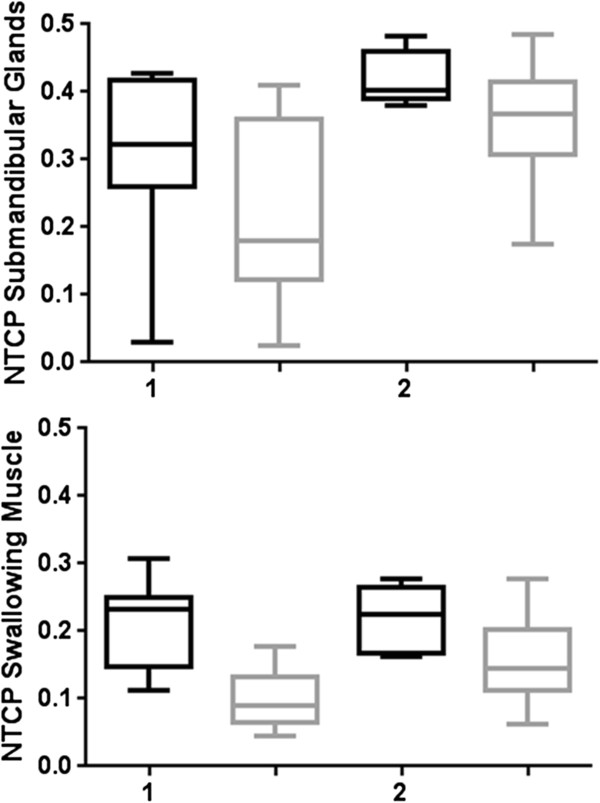
**NTCP-values for the swallowing muscles and the submandibular glands for laryngeal cancer patients (1) Patients without positive nodes, (2) Patients with positive nodes.** NTCP values for tight margins (black boxes) were statistically significantly lower than those for clinical margins (grey boxes).

**Figure 5:**
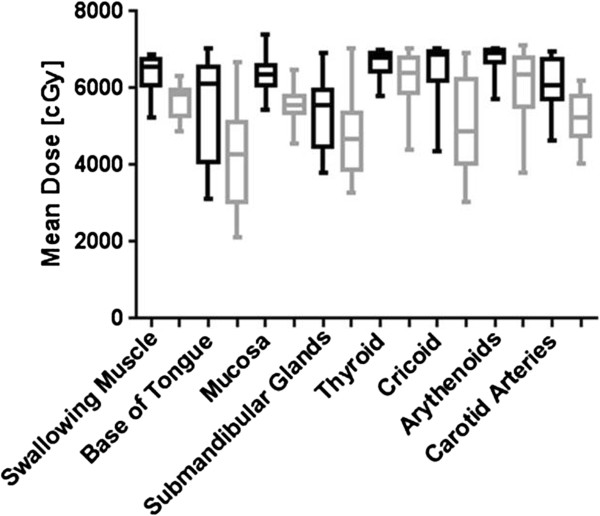
**Mean dose reduction.** Mean dose values for tight margins (black boxes) were statistically significantly lower than those for clinical margins (grey boxes).

The relative difference in dose between clinical and tight margins was compared. A reduction in mean dose and NTCP-value was found for all patients independently of TNM-stage.For patients without positive nodes, the NTCP decreased by 53% for the swallowing muscles and by 23% for the submandibular glands (Figure 4). When positive lymph nodes were present, the NTCP decreased with respectively 29% and 15%.The reduction in mean dose over all OARs was 16% in group 1 versus 11% in group 2 (Figure 5). The differences between median dose values and between NTCP values were all statistically significant.All OARs, except of the thyroid and the arytenoids, received a mean dose lower than 60 Gy using tight margins (Figure 4). Using conservative margins, no OARs in group 2 and only the base of the tongue and the submandibular glands in group 1 received a mean dose lower than 60 Gy. Largest mean dose reduction was observed in the base of the tongue and the cricoid.

## Discussion

Using our clinical margins, the PTV for laryngeal tumors was about 10 times as large as the GTV. The PTV might be reduced with 50% when evidence based tight margins are applied. This volume reduction was advantageous for all delineated OARs independent of the presence of (elective) nodal volumes.

Elective treatment of the lymph node levels II to IV results in a large volume receiving a high radiation dose [6, 13]. Improved knowledge on recurrence patterns or detailed lymph node imaging studies might facilitate more specific delineation of nodal regions which will result in a larger gain using tight margins for laryngeal carcinoma.

The relative importance of the various anatomical structures and substructures in causing radiation toxicity is the subject of current research [3, 4, 23]. Therefore, various OARs were analysed in this study. Toxicity was estimated in two ways: mean dose and NTCP. The constraint of mean dose lower than 60 Gy was analyzed since it has been shown that a dose limitation of 60 Gy to the larynx results in a low risk of aspiration [9].

The NTCP is the best estimate of complication risk. However dose response models of most OARs in the head-and-neck region are currently not available since OARs in this region were historically homogeneously irradiated.

Several studies have been performed that show a dose-volume-effect of the swallowing muscles for dysphagia. However, a clinically relevant dose response model obtained in a large patient population is lacking [23, 24]. Levendag et al. proposed a subdivision of the swallowing muscles in five structures and published a dose response curve for the cranial part of the swallowing muscles [25]. The use of this model will result in a larger gain in NTCP than using the model of Jackson based on the total volume of the swallowing muscles [21]. We decided to define the swallowing muscles as one structure, since the exact contribution of the different structures to radiation induced complication has not been fully understood.

The largest reduction in mean dose was observed in the base of the tongue and the cricoid. These structures are most of the time located outside of the target volume when tight margins are used.

When elective lymph node regions are included, a simultaneous integrated boost (SIB) technique may give better organ sparing than a separate consecutive boost due to beneficial dose distribution. However a SIB treatment will lead to increased toxicity due to high fraction doses in normal tissue within the high dose region.

In this work we translated new insight and techniques applied for laryngeal cancer irradiation to optimized treatment margins and quantified the clinical benefits. At our department this work has resulted in a reduction of the CTV-PTV margin to 3 mm and a reduction of the margin applied for internal motion. For a reduction of the GTV-CTV margin more results from the pathology studies are awaited.

Although definitive guidelines for laryngeal tumor delineation and margins cannot be given, the results from this study indicate that further research in this area is needed and might result in significantly reduced toxicity.

## Conclusion

Applying tight margins around the primary tumor in laryngeal carcinoma theoretically resulted in 45% less late toxicity for the swallowing muscles and 20% for the submandibular glands compared to conservative margins, independent of treatment of the lymph node levels II to IV. Therefore it is worth to translate the results from image validation, microscopic extension and tumor motion studies into guidelines for evidence based tight margins around the primary tumor of supraglottic laryngeal cancer patients which can be implemented in clinical practice.
